# Comparison of bispectral index and patient state index during general anesthesia with remimazolam

**DOI:** 10.1186/s12871-025-03464-6

**Published:** 2025-11-17

**Authors:** Takuya Shiraishi, Musashi Yahagi, Masashi Yoshida, Manami Yawata, Haruka Kurokawa, Yamato Tamakawa, Yasuaki Kawakita, Yuichi Yaguchi

**Affiliations:** Department of Anesthesiology, Hitachi General Hospital, 2-1-1 Jonan-cho, Hitachi, Ibaraki 317-0077 Japan

**Keywords:** General anesthesia, Remimazolam, Bispectral index, Patient state index, Electroencephalography, Monitoring

## Abstract

**Background:**

Processed electroencephalographic indices, such as the Bispectral Index (BIS) and Patient State Index (PSI), are widely used to assess anesthetic depth; however, their comparative behavior under remimazolam remains unclear. In practice, either index may deviate from its target range despite stable anesthesia, and the response of the companion monitor is uncertain. This study examined whether the two indices moved in parallel or diverged when one departed from its nominal range during remimazolam anesthesia.

**Methods:**

In this prospective, single-center study, BIS and PSI were simultaneously recorded at 21 predefined time points from induction to emergence in 60 adults who received remimazolam-based general anesthesia. Remimazolam was induced at 12 mg/kg/h and maintained at 1 mg/kg/h; all patients received neuromuscular blockade (train-of-four ≤ 1). Two trained observers captured synchronized monitor screenshots and documented the Signal Quality Index, electromyography (EMG), and suppression ratio. Analyses used 994 paired observations: pooled Pearson and repeated-measures correlations, simple linear regression (PSI vs. BIS), repeated-measures Bland–Altman analysis, and a linear mixed-effects model for BIS–PSI differences, including anesthetic phase and EMG as fixed effects and patient as a random intercept.

**Results:**

BIS and PSI showed a strong correlation (*r* = 0.89) and linear relationship (PSI = 0.936 × BIS − 7.26; R² = 0.79). The repeated-measures Bland–Altman bias was + 10.9 (95% CI 10.3–11.4) with 95% limit of agreement (LoA) − 7.8 to 29.5. Phase-wise modeling showed minimal discrepancies while awake and larger differences during intubation and emergence (Tukey-adjusted *p* < 0.001), even after EMG adjustment. Both indices tracked the anesthetic depth in parallel but were not numerically interchangeable.

**Conclusions:**

During remimazolam anesthesia, BIS and PSI exhibited parallel temporal trends, indicating shared cortical dynamics despite distinct algorithms. When either index deviated, either elevated or suppressed, the other generally moved in the same direction, reflecting consistent responsiveness rather than device artifacts. Phase-dependent discrepancies and wide agreement limits suggest that each index should be interpreted within its reference framework. Understanding this “parallel yet nonidentical” behavior may support context-aware, device-specific depth monitoring.

**Trial registration:**

University Hospital Medical Information Network Clinical Trials Registry (UMIN-CTR, UMIN000058212; registered on June 18, 2025).

**Supplementary Information:**

The online version contains supplementary material available at 10.1186/s12871-025-03464-6.

## Introduction

An accurate assessment of anesthetic depth is crucial for preventing intraoperative awareness and excessive sedation, both of which are associated with adverse postoperative outcomes [1–4]. Electroencephalography (EEG)–based monitors have become integral to this task because they can continuously and noninvasively measure brain electrical activity across levels of consciousness. Among these tools, the Bispectral Index (BIS) and the Patient State Index (PSI) are widely used in clinical practice to estimate the level of consciousness during general anesthesia [5–7].

The BIS has been extensively used in clinical practice for many years, whereas the PSI is a more recently developed index that employs a proprietary algorithm based on a four-channel EEG input [7, 8]. Although both indices estimate anesthetic depth from EEG signals, differences in algorithmic processing and electrode configuration can lead to systematic discrepancies, particularly under conditions of high electromyographic (EMG) activity or poor signal quality [7].

Several studies have assessed the concordance between BIS and PSI values across different anesthetic agents. Moderate-to-high correlations have been reported under both sevoflurane and propofol, particularly during maintenance and emergence [9–11]. In some reports, BIS showed a slightly stronger association with MOAA/S scores and features of raw EEG, especially during emergence from propofol anesthesia [11]. A study on desflurane also found a moderate correlation during the intraoperative and emergence periods; however, the generalizability was limited by the small sample size (*n* = 20) and heterogeneity in patient characteristics and anesthetic protocols [12]. Collectively, differences in agents, monitoring phases, and populations constrain direct comparisons and their clinical applicability.

Remimazolam, an ultrashort-acting benzodiazepine with favorable pharmacokinetics and hemodynamic stability, has gained traction in clinical anesthesia [13–16]. However, EEG-based monitoring data under remimazolam remain limited, with most reports focusing on sedation or single-bolus protocols [17, 18]. For example, in elderly men undergoing transurethral resection of the prostate under spinal anesthesia, BIS and PSI after a single remimazolam bolus were strongly correlated (*r* = 0.796), but BIS correlated more closely with MOAA/S than PSI (*r* = 0.568 vs. 0.390) [18]. However, the single-dose design and highly specific population limit extrapolation to general anesthesia.

Notably, the recommended numerical ranges for processed EEG indices have not been specifically validated for remimazolam. In routine clinical practice, clinicians occasionally encounter situations where either the processed EEG index (BIS or PSI) deviates from its expected target range despite apparently stable anesthesia. Whether the companion monitor shows a similar deviation or remains within the range is often uncertain, particularly under remimazolam, whose algorithm-specific behavior has not been well defined. Clarifying how the two indices respond when one departs from its nominal range—whether they move in parallel or diverge—would provide practical insight into interpreting depth monitoring under this agent.

To address these gaps, we simultaneously monitored the BIS and PSI during remimazolam-based general anesthesia. We summarized trajectories across predefined, clinically annotated perioperative time points spanning induction to emergence and evaluated the overall association, linear mapping, and measurement agreement between the indices. We further examined phase-specific differences while accounting for EMG activity and signal quality using linear mixed-effects models.

We hypothesized that the BIS and PSI would correlate strongly but exhibit systematic level differences that vary by anesthetic phase and EMG burden, with larger discrepancies expected during rapidly changing conditions.

## Methods

### Study design and participants

This prospective, single-center observational study was conducted at Hitachi General Hospital (June–October 2025). Adults aged 20–60 years who were scheduled for elective surgery under general anesthesia were enrolled. The exclusion criteria were as follows: American Society of Anesthesiologists physical status (ASA PS) III or higher, history of cerebrovascular disease, current use of benzodiazepines, dermatologic conditions on the forehead that precluded sensor adhesion, and a history of psychiatric disorder. The protocol was approved by the Institutional Review Board (approval No. 2024 − 101), written informed consent was obtained from all participants, and the trial was registered at UMIN-CTR (UMIN000058212).

The target sample size was 60. This pragmatic target was set a priori based on planning for the primary association analyses: assuming a moderate BIS–PSI association (*r* ≈ 0.6–0.7) and ≥ 10 paired observations per participant across predefined perioperative time points, standard precision planning (Fisher z–based) indicated that a sample of approximately 54 participants would yield an acceptably narrow two-sided 95% confidence interval for the pooled correlation (width around 0.30) while also supporting stable estimation of repeated-measures Bland–Altman limits under a mixed-effects framework. Allowing for up to ~ 10% attrition or unusable data, we judged that 60 participants would be sufficient.

### Perioperative monitoring and data acquisition

Standard monitoring [electrocardiography (ECG), noninvasive blood pressure, and peripheral oxygen saturation (SpO₂)] was initiated upon arrival in the operating room. BIS and PSI sensors were applied simultaneously on the forehead throughout the anesthesia. For clarity, the BIS sensors were placed just above the eyebrows, and the PSI sensors were positioned immediately superior to the forehead (without overlap).

Two trained research staff members, independent of anesthetic care, captured synchronized screenshots from both monitors at each predefined moment and transcribed the corresponding values to a standardized case report form. The Signal Quality Index (SQI), electromyography (EMG), and suppression ratio (SR) were recorded. Each entry was double-checked against the screenshots taken at the time of recording.

Sensor impedance and visible artifacts were checked at each scheduled recording, and the sensors were repositioned when necessary to maintain data quality.

### Anesthesia management

General anesthesia was induced with remimazolam at 12 mg/kg/h. After loss of consciousness (LOC), defined as a Modified Observer’s Assessment of Alertness/Sedation (MOAA/S) score ≤ 1, assessed by a trained investigator, the infusion rate was reduced to 1 mg/kg/h for maintenance. Remifentanil was initiated at 0.25 µg/kg/min and titrated according to clinical requirements. All patients received a nondepolarizing neuromuscular blocker, and neuromuscular blockade was titrated to maintain a train-of-four (TOF) count ≤ 1 throughout intubation and maintenance, with supplemental dosing as clinically indicated.

### Time points and clinical phases

The BIS and PSI were recorded at 21 predefined time points spanning induction to emergence.

- T0: awake baseline.
- T1: LOC after remimazolam.
- T2: immediately after tracheal intubation.
- T3–T5: 5, 10, 15 min after intubation and before skin incision.
- TI: at skin incision.
- T15–T180: every 15 min after the start of surgery.
- TE: at tracheal extubation.
- TX: defined from the outset as the instant the attending anesthesiologist formally declared the patient “ready for operating-room exit” after verifying hemodynamics, consciousness, analgesia, and ventilation.

For phase-wise analyses, time points were grouped a priori as follows: Awake (T0), LOC (T1), Intubation (T2), Pre-incision (T3–T5), Intraoperative (TI, T15–T180), and Emergence (TE, TX). If the surgery ended before T180, recording was stopped at the last available point.

### Outcomes

The primary objective was to characterize the relationship and numerical agreement between BIS and PSI across the perioperative period. The prespecified primary outcomes were as follows: (i) overall association between paired BIS and PSI values assessed by pooled Pearson correlation with cluster-aware 95% confidence intervals, supplemented by repeated-measures correlation (rmcorr); (ii) linear mapping, modeling PSI as a function of BIS using simple linear regression; and (iii) agreement, quantified by Bland–Altman bias (BIS − PSI) and 95% limits of agreement (LoA) estimated using a repeated-measures approach.

The secondary outcomes were time-course summaries (mean ± SD at each predefined time point) and phase-wise differences in BIS − PSI, with EMG considered as described below.

### Statistical analysis

Continuous values are presented as mean ± SD unless stated. BIS and PSI were paired at each time point, and analyses used all available paired observations.

The overall association between BIS and PSI was assessed using pooled Pearson correlation across all paired observations, with 95% confidence intervals obtained using patient-level cluster bootstrap to account for repeated measures; a repeated-measures correlation (rmcorr) was additionally estimated.

A simple linear regression was used to model the PSI as a function of BIS, with cluster-robust standard errors to account for within-subject clustering. We report the regression equation, slope/intercept 95% confidence intervals and R².

Numerical agreement was examined using the Bland–Altman method for repeated measurements [19]. The mean bias (BIS–PSI) and 95% LoA were derived from a mixed-effects formulation with random intercepts for subjects, combining within- and between-subject variance components.

To evaluate the phase effects on the discrepancy, we fitted a linear mixed-effects model with D = (BIS − PSI) as the dependent variable, anesthetic phase (six levels) as a fixed effect, and patient as a random intercept. High EMG was incorporated as a prespecified binary covariate, defined as BIS-EMG ≥ 40 (as displayed by the monitor) or the presence of a numeric EMG readout on the PSI monitor at that time point. Planned pairwise contrasts between phases were obtained from the model using marginal means with Tukey’s adjustment. Because neuromuscular blockade (NMB) was applied uniformly (TOF ≤ 1 in all cases), subgrouping by the presence or absence of NMB was not feasible; instead, we modeled muscle activity directly via EMG as a pre-specified covariate to capture the downstream impact of residual neuromuscular transmission on the index behavior.

Graphics were created with ggplot2; all tests were two-sided with *p* < 0.05 considered statistically significant. Analyses were conducted in R (version 4.5.1; R Foundation for Statistical Computing) using standard packages for mixed-effects modeling, cluster-robust inference, and repeated-measures correlation.

## Results

### Patient characteristics

Sixty adults (20 male/40 female) were analyzed. Mean age was 48.9 ± 8.5 years; height 160.6 ± 9.8 cm; weight 63.5 ± 14.0 kg. ASA-PS grades I and II were observed in 33 (5%) and 27 (45%) patients, respectively. The procedures included gynecological (*n* = 17), breast (*n* = 12), gastrointestinal (*n* = 20), urological (*n* = 1), orthopedic (*n* = 4), thoracic (*n* = 1), and other (*n* = 5) surgeries. The baseline details are presented in Table 1.

**Table 1:** Patient characteristics (*n* = 60)

Variable	*n* (%) or Mean ± SD
Age (years)	48.9 ± 8.5
Height (cm)	160.6 ± 9.8
Weight (kg)	63.5 ± 14.0
Sex	
Male	20 (33)
Female	40 (67)
ASA-PS	
I	33 (55)
II	27 (45)
Comorbidities	
Hyperlipidemia	6 (10)
Hypertension	5 (8)
Smoker	5 (8)
Obesity	5 (8)
Diabetes mellitus	4 (7)
Glaucoma	2 (3)
Rheumatoid arthritis	1 (2)
Obstructive ventilatory disorder	1 (2)
Asthma	1 (2)
Atrial fibrillation	1 (2)
Surgical category	
Gynecological	17 (28)
Breast	12 (20)
Gastrointestinal surgery	20 (33)
Urology	1 (2)
Orthopedic	4 (7)
Thoracic	1 (2)
Other	5 (8)

*Abbreviations:* *ASA-PS* American Society of Anesthesiologists physical status, *SD* standard deviation

### Time-course comparison of BIS and PSI

**Fig. 1:**
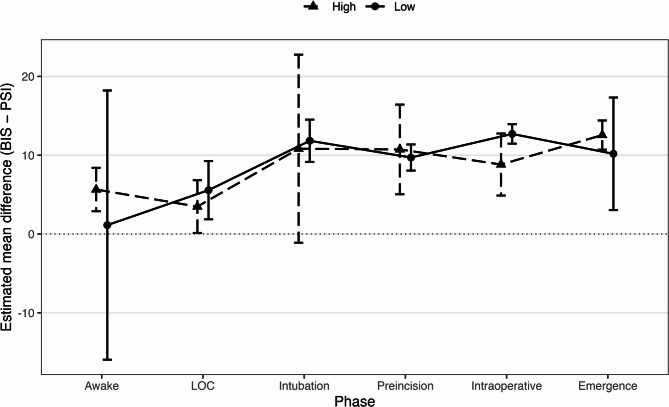
Perioperative time course of BIS and PSI across predefined event-aligned time points. Legend: Data are presented as means ± standard error (SE) of paired BIS and PSI at each predefined perioperative time point (T0–TX). The x-axis shows equally spaced event-aligned time points for clarity; the intervals do not represent proportional elapsed time. Abbreviations: BIS = Bispectral Index; PSI = Patient State Index; SE = standard error; LOC = loss of consciousness; TI = skin incision; TE = tracheal extubation; TX = operating room exit

Across the perioperative period, both indices decreased after induction, remained stable during maintenance, and increased at emergence (Fig. 1; Table 2). The BIS values were consistently higher than the PSI values at all time points. For example, during maintenance at T120, BIS was 48.9 ± 9.9 and PSI 35.6 ± 6.6; at extubation (TE), BIS and PSI were 88.3 ± 12.3 and 75.9 ± 11.6, respectively. Numerical summaries at all 21 predefined time points are provided in the Supplementary Table S1.

**Table 2:** Summary of BIS, PSI, and related EEG parameters at each anesthetic phase

Time	*n* (pairs)	BIS (Mean ± SD)	PSI (Mean ± SD)	EMG-BIS (Mean ± SD)	EMG-PSI (Mean ± SD)	SQI (Mean ± SD)
T0	60	93.5 ± 9.4	90.8 ± 6.6	47.9 ± 5.3	24.2 ± 25.2	51.5 ± 22.8
T1	60	73.6 ± 15.1	68.3 ± 16.5	41.3 ± 7.5	7.7 ± 15.9	80.6 ± 11.0
T2	60	49.3 ± 10.3	37.8 ± 8.7	31.2 ± 13.5	0.3 ± 1.8	87.2 ± 9.0
T3	60	51.3 ± 10.3	41.1 ± 9.5	27.7 ± 14.0	0.2 ± 0.9	89.7 ± 11.8
T4	60	50.1 ± 10.3	40.2 ± 8.8	27.0 ± 14.4	0.5 ± 1.9	92.9 ± 8.3
T5	60	48.5 ± 9.5	38.8 ± 9.5	27.5 ± 16.5	0.2 ± 1.4	91.6 ± 11.2
TI	60	48.0 ± 9.0	37.5 ± 8.0	26.4 ± 17.0	0.1 ± 1.2	85.5 ± 14.8
T15	60	46.2 ± 9.1	35.5 ± 9.1	25.5 ± 17.1	0.7 ± 2.4	82.0 ± 16.7
T30	60	46.4 ± 9.1	34.6 ± 8.0	24.2 ± 15.4	1.0 ± 4.3	83.2 ± 17.7
T45	60	47.6 ± 9.3	34.9 ± 8.0	23.7 ± 14.4	0.6 ± 3.3	84.4 ± 15.2
T60	54	48.0 ± 9.0	34.6 ± 7.8	24.4 ± 14.0	0.3 ± 1.6	87.7 ± 12.5
T75	46	47.7 ± 10.5	33.6 ± 8.7	25.2 ± 14.3	0.5 ± 2.6	87.9 ± 12.2
T90	38	48.5 ± 9.2	35.5 ± 7.3	24.7 ± 13.8	1.4 ± 6.1	89.8 ± 10.0
T105	30	50.0 ± 11.8	35.8 ± 9.6	23.7 ± 14.2	1.0 ± 5.7	87.4 ± 12.0
T120	28	48.9 ± 9.9	35.6 ± 6.6	23.8 ± 14.5	0.7 ± 3.8	90.1 ± 11.7
T135	24	49.2 ± 9.4	36.3 ± 7.7	22.6 ± 13.9	0.0 ± 0.0	88.7 ± 12.4
T150	22	50.7 ± 10.3	38.1 ± 7.8	23.5 ± 15.4	0.9 ± 3.4	86.8 ± 18.8
T165	17	50.2 ± 8.5	37.3 ± 7.1	21.9 ± 15.0	0.4 ± 1.5	88.6 ± 15.6
T180	15	48.4 ± 9.3	36.2 ± 6.4	23.3 ± 15.1	1.1 ± 4.4	92.1 ± 10.8
TE	60	88.3 ± 12.3	75.9 ± 11.6	48.9 ± 8.7	35.0 ± 27.8	67.8 ± 15.0
TX	60	93.5 ± 6.2	80.5 ± 12.1	48.5 ± 7.2	37.3 ± 27.4	65.2 ± 19.0

Data are presented as mean ± SD for each time point during general anesthesia with remimazolam. T0 = awake (baseline before induction), T1 = loss of consciousness, T2 = tracheal intubation, T3–T5 = pre-incision, TI–T180 = intraoperative period, TE–TX = emergence phase

The values represent 60 paired observations. These descriptive statistics were used to illustrate the temporal trends of BIS and PSI across the anesthetic phases, providing context for the correlation and Bland–Altman analyses (Tables 3 and 4)

*Abbreviations: BIS * Bispectral Index, *PSI* Patient State Index, *EMG* electromyography activity (%), *SQI* signal quality index (%)

### Correlation analysis

**Fig. 2:**
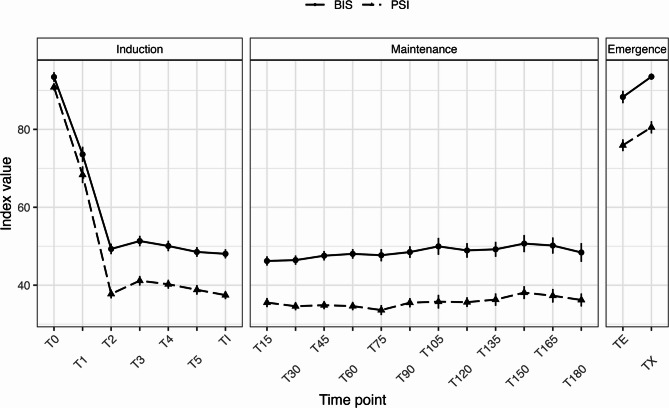
Association and linear mapping between PSI and BIS. Legend: Each marker represents a paired BIS–PSI observation pooled across predefined perioperative time points. The solid line depicts the simple linear regression fit (PSI ~ BIS) with the 95% confidence band. The overall association was quantified by pooled Pearson correlation and repeated-measures correlation (rmcorr), as described in the Methods section. Abbreviations: BIS = Bispectral Index; PSI = Patient State Index; CI = confidence interval; rmcorr = repeated-measures correlation

Pooling all paired observations (*n* = 994), BIS and PSI showed a strong positive correlation (pooled Pearson *r* = 0.89, 95% CI 0.88–0.90; *p* < 0.001; Fig. 2). The repeated-measures correlation (rmcorr) similarly indicated a high within-subject association (*r* = 0.905, 95% CI 0.892–0.916; *p* < 0.001) (Fig. 2; Table 3).

**Table 3:** Correlation and regression analyses between BIS and PSI during remimazolam anesthesia

*n* (pairs)	Pearson *r* (95% CI)	Pearson *p*	Slope (95% CI)	Intercept (95% CI)	*R*²	rmcorr *r* (95% CI)	Rmcorr *p*
994	0.89 (0.88–0.90)	*p* < 0.001	0.936 (0.907–0.966)	−7.26 (−9.09–−5.43)	0.79	0.905 (0.892–0.916)	*p* < 0.001

Data are presented as summary statistics for the overall dataset (n = 994 paired observations) during general anesthesia with remimazolam

Pearson’s correlation coefficients (r), corresponding 95% confidence intervals (CI), and simple linear regression parameters (slope, intercept, and coefficient of determination R²) were calculated to assess the overall association between BIS and PSI

A repeated-measures correlation (rmcorr) was computed to account for within-subject clustering across multiple time points.

These analyses quantified the degree of linear association and consistency between the two EEG-derived indices, providing a statistical foundation for the agreement analysis in Table 4

*Abbreviations:* *BIS* Bispectral Index, *PSI* Patient State Index, *rmcorr* repeated measures correlation, *CI* confidence interval

### Linear regression analysis between BIS and PSI

Simple linear regression demonstrated a robust linear relationship (Fig. 2): PSI = 0.936 × BIS − 7.26, with R² = 0.79. The 95% CIs were 0.907–0.966 for the slope and − 9.09 to − 5.43 for the intercept, respectively. Although an average mapping exists, the wide LoA preclude pointwise substitution of one index for the other.

### Agreement analysis using the Bland–Altman plot

**Fig. 3:**
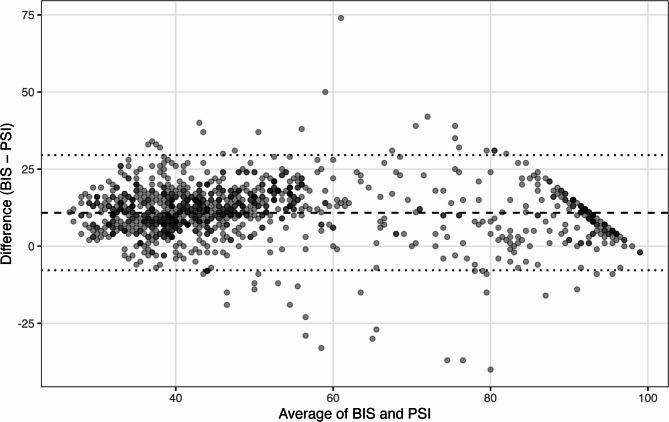
Repeated-measures Bland–Altman analysis of BIS and PSI. Legend: The difference (BIS−PSI) is plotted against the average ((BIS+PSI)/2) for each paired observation. The solid horizontal line indicates the estimated mean bias, and the dashed lines show the 95% limits of agreement (LoA), both derived from a repeated-measures Bland–Altman approach that accounts for within-subject clustering via a mixed-effects formulation. Positive values indicate a higher BIS relative to the PSI. Abbreviations: BIS = Bispectral Index; PSI = Patient State Index; LoA = limits of agreement

A repeated-measures Bland–Altman analysis yielded a mean bias (BIS − PSI) of + 10.9 (95% CI 10.3–11.4) with 95% LoA − 7.8 to 29.5 (95% CI − 8.5 to 30.3), indicating that, despite the strong correlation, the indices are not numerically interchangeable (Fig. 3; Table 4). Positive values indicate a higher BIS relative to the PSI.

**Table 4:** Repeated-measures Bland–Altman analysis between BIS and PSI values

*n* (pairs)	Bias (BIS − PSI) ± SD	95% CI for Bias	LoA (Bias ± 1.96 × SD)	95% CI for LoA	Between-subject SD	Within-subject SD
994	10.9 ± 9.5	10.3–11.4	−7.8–29.5	−8.5–30.3	3.7	8.8

Data are presented as results of the repeated-measures Bland–Altman analysis for BIS–PSI differences across all paired observations (n = 994)

Bias represents the mean difference (BIS − PSI) ± standard deviation (SD), with corresponding 95% confidence intervals (CI) for the bias and limits of agreement (LoA)

The between-subject and within-subject variance components were estimated to account for repeated measurements within the individuals.

Positive values indicated higher BIS readings relative to the PSI

This analysis quantified the overall agreement between the two EEG-derived indices while adjusting for within-subject clustering

*Abbreviations:* *BIS* Bispectral Index, *PSI* Patient State Index, *LoA* limits of agreement, *SD* standard deviation, *CI* confidence interval

### Discrepancy between BIS and PSI across clinical event phases

**Fig. 4:**
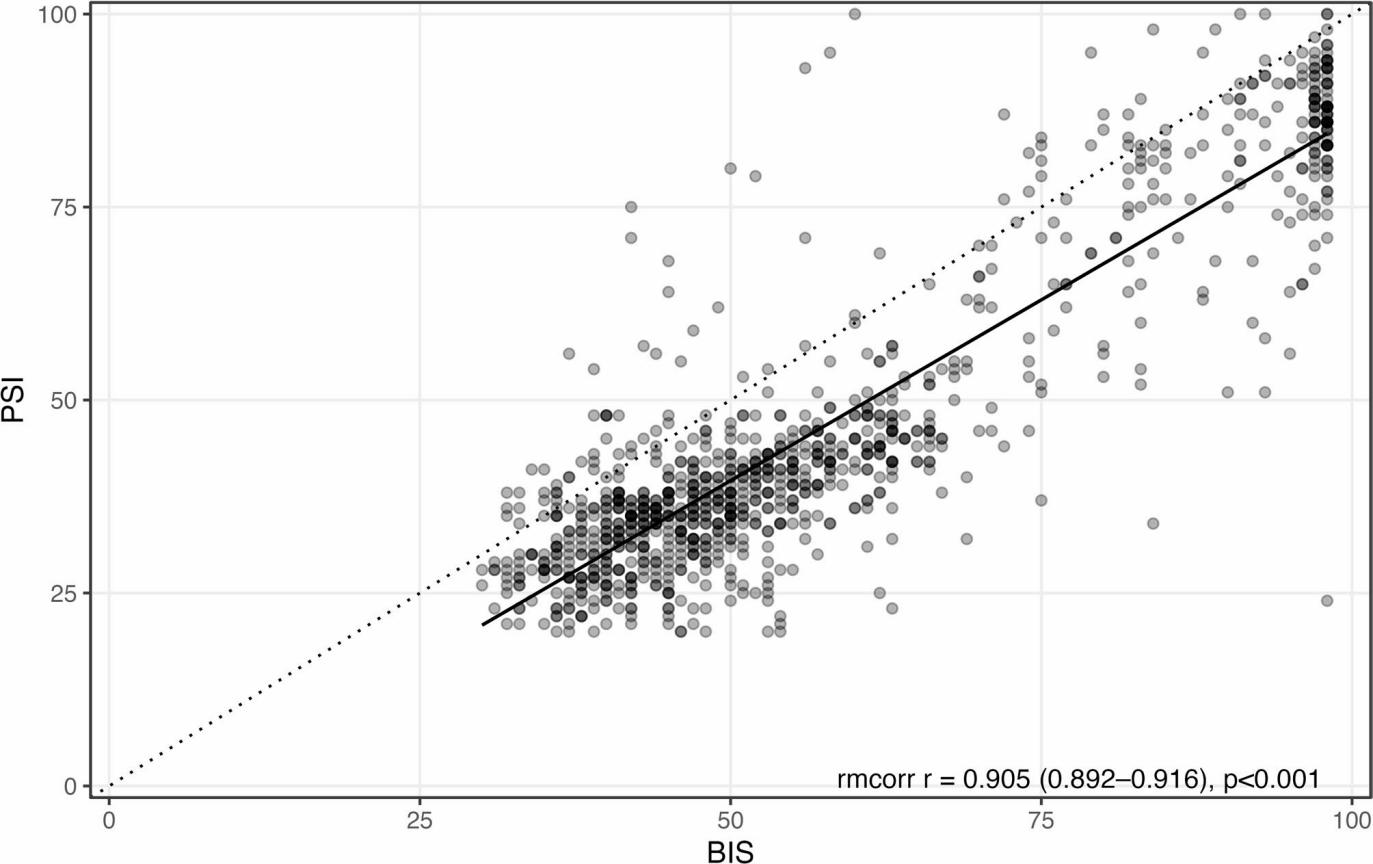
Phase-specific BIS–PSI differences conditional on EMG (LME). Legend: Data are presented as estimated marginal means (EMMs) ± 95% confidence intervals (CI) from a linear mixed-effects model (LME) with anesthetic phase and electromyography (EMG) as fixed effects and patient as a random intercept. Positive values indicate a higher BIS relative to the PSI. High EMG was predefined as BIS-EMG ≥ 40 or the presence of a numeric EMG readout on the PSI monitor. These results illustrate phase-dependent differences and EMG-related modulation of BIS–PSI discrepancies. Abbreviations: BIS = Bispectral Index; PSI = Patient State Index; EMG = electromyography; EMM = estimated marginal mean; CI = confidence interval; LME = linear mixed-effects model

Using a linear mixed-effects model with the anesthetic phase and EMG as fixed effects, the BIS–PSI difference varied by phase. EMG-averaged, Tukey-adjusted pairwise comparisons showed larger discrepancies vs. Awake for Intubation, Pre-incision, Intraoperative, and Emergence (all *p* < 0.001), whereas LOC vs. Awake was not significant (*p* = 0.615). Among the non-awake phases, Intraoperative exceeded Pre-incision (Δ = 2.5 [0.3–4.8], *p* = 0.017) (Fig. 4). Phase-wise estimated marginal means (EMMs) stratified by EMG are summarized in Table 5, and Tukey-adjusted contrasts are provided in Supplementary Tables S2 (EMG strata) and S3 (EMG-averaged main effect).

**Table 5:** Estimated marginal means of BIS–PSI differences across anesthetic phases stratified by EMG activity

Phase	EMG	Estimate [95% CI]
Awake	Low	1.1 [−16.0–18.2]
Awake	High	5.6 [2.9–8.4]
LOC	Low	5.6 [1.9–9.3]
LOC	High	3.5 [0.1–6.8]
Intubation	Low	11.8 [9.2–14.5]
Intubation	High	10.8 [−1.1–22.8]
Preincision	Low	9.7 [8.0–11.4]
Preincision	High	10.7 [5.0–16.4]
Intraoperative	Low	12.7 [11.5–13.9]
Intraoperative	High	8.8 [4.9–12.8]
Emergence	Low	10.2 [3.0–17.3]
Emergence	High	12.6 [10.7–14.4]

Data are presented as estimated marginal means (EMMs) ± 95% confidence intervals (CI) derived from a linear mixed-effects model (LME), including the anesthetic phase and electromyography (EMG) activity as fixed effects

Positive values indicate a higher BIS relative to the PSI

These results illustrate the phase-specific trend and EMG-related modulation of BIS–PSI differences, complementing the pairwise contrasts in Supplementary Table S2 and the graphical depiction in Fig. 4

*Abbreviations:* *BIS* Bispectral Index, *PSI* Patient State Index, *EMG* electromyography activity, *EMM* estimated marginal mean, *CI* confidence interval, *LME* linear mixed-effects model

## Discussion

### Comparison between BIS and PSI during remimazolam anesthesia

This prospective observational study of 60 adults characterized the temporal relationship and numerical agreement between BIS and PSI under remimazolam-based general anesthesia. Across 21 predefined time points, BIS values were consistently higher than PSI values, with an overall mean bias of + 10.9 and 95% LoA − 7.8 to 29.5 from a repeated-measures Bland–Altman analysis. Despite this systematic offset, the indices exhibited a strong positive correlation (*r* = 0.89) and a stable linear association (PSI = 0.936 × BIS − 7.26; R² = 0.79), indicating parallel tracking with limited interchangeability and extending prior observations under sevoflurane and propofol to remimazolam [11, 20]. Clinically, this parallel movement addresses a common concern: when the BIS appears higher than expected under remimazolam, the PSI generally moves in the same direction, suggesting shared cortical dynamics rather than device-specific artifacts. Earlier investigations have also demonstrated strong BIS–PSI correlations across agents despite modest numeric offsets [21, 22]. Phase-wise mixed-effects modeling further showed that the BIS–PSI gap was smallest while awake and larger around intubation and emergence—epochs prone to myogenic activity; differences versus Awake were significant for Intubation, Pre-incision, Intraoperative, and Emergence (Tukey-adjusted), whereas LOC vs. Awake was not. The Intraoperative >Pre-incision contrast also emerged (EMG-averaged), and these phase effects persisted after accounting for EMG, indicating both artifact-related and phase-dependent components in the between-index discrepancy.

### Technical differences between BIS and PSI

The BIS and PSI are EEG-derived indices that differ in spatial sampling and signal processing. The BIS summarizes frontal EEG with a relatively greater weighting of higher-frequency (β) components and is susceptible to EMG contamination, particularly during stimulation or increased muscle tone [23, 24]. The PSI, computed from a four-channel frontal montage (SedLine^®^), incorporates proprietary artifact-suppression approaches; its Artifact Index (ARTF) estimates non-cortical contamination, including EMG and electrocardiography (ECG) [8, 25]. These design features plausibly explain our observation that the BIS–PSI gap widened at intubation and emergence, often accompanied by increased EMG, while the overall trajectories remained parallel, suggesting shared cortical dynamics with device-specific sensitivity superimposed [26–28].

Not all high-EMG periods showed marked dissociation, implying that divergence depends on EMG magnitude/source and signal quality; thus, reviewing EMG/SQI alongside index values is advisable. Under remimazolam, benzodiazepine-linked β/σ augmentation may also differentially affect BIS weighting relative to PSI, potentially contributing to the observed offset [30]. Both indices rely on proprietary, undisclosed algorithms; only high-level descriptions are publicly available for BIS and PSI, which precludes algorithm-level interpretation of divergences.

### Clinical implications

Our findings demonstrate that BIS and PSI generally track in parallel during remimazolam anesthesia, addressing the practical question of whether both monitors respond similarly when BIS appears high in routine practice. Because index targets for remimazolam have not been established, our results clarify that the two devices move in parallel but diverge numerically, particularly during EMG-prone or rapidly changing phases, underscoring the need to interpret each monitor within its own reference framework. While different nominal target ranges (e.g., BIS 40–60 vs. PSI 25–50 under volatile/propofol anesthesia) can partly explain level offsets, our phase-specific analyses and wide repeated-measures LoA indicate more than a fixed numerical shift, particularly during EMG-active or rapidly changing states. Hence, direct substitution remains unsafe when remimazolam is used.

Our data support three practical implications. First, BIS and PSI should not be used interchangeably; the recommended numeric ranges differ, and cross-device overlap is limited [20–22]. Second, clinicians should prioritize trends and context over single absolute values: (i) check EMG/SQI and electrode contact before adjusting hypnotic dosing; (ii) corroborate with hemodynamics, movement, and surgical stage; (iii) beware of EMG-related BIS elevations during stimulation or spontaneous breathing; and (iv) consider multimodal monitoring when artifact risk is high [29]. Third, PSI may appear numerically more stable under muscle activity due to artifact suppression, whereas BIS can show upward excursions despite a stable cortical state; conversely, stronger artifact filtering might render PSI less responsive to subtle cortical transitions in some contexts. For remimazolam, PSI target ranges remain undefined; until index-specific thresholds are established, each device should be interpreted within its own reference framework, integrated with clinical signs [25, 30–32].

### Methodological strengths

The design features enhanced the internal validity. We used a standardized induction (12 mg/kg/h) and fixed maintenance (1 mg/kg/h) in all cases, limiting the between-patient variability. Simultaneous BIS/PSI acquisition at 21 event-aligned time points captured dynamic transitions and steady states with high temporal resolution. The dataset contained 994 paired observations, enabling precise estimation of the correlation, regression, and repeated-measures Bland–Altman statistics. Importantly, two trained research staff members obtained synchronized screenshots at predefined moments and double-checked transcribed values while recording EMG, SQI, and SR, which supported artifact awareness and contextual interpretation of device divergence.

### Limitations and future directions

This study has several limitations. First, although we addressed within-subject dependence using repeated-measures LoA and mixed-effects models, residual clustering and model-assumption misspecification may still influence estimates. In addition to EMG-related effects, index-specific latency arising from algorithmic computation and pharmacokinetic/pharmacodynamic dynamics may attenuate differences near the LOC and accentuate discrepancies during rapid transitions at emergence. This mechanism likely contributes to the phase-dependent patterns observed in the present study.

Second, the single-center cohort of relatively healthy adults (ASA I–II) limits the generalizability to older, frailer, or neurologically vulnerable populations and other practice settings.

Third, we did not analyze raw EEG; phenomena such as burst suppression, β/σ dynamics, or micro-arousals were not interrogated beyond processed indices, and both devices rely on proprietary transformations, precluding algorithm-level inspections.

Fourth, our fixed remimazolam maintenance reduced exposure heterogeneity; future studies should test alternative infusion strategies and pharmacokinetic/pharmacodynamic (PK/PD)-integrated titration, linking effect-site estimates to processed EEG trajectories. We did not prespecify time-aligned stratification by the depth of neuromuscular block beyond the uniform TOF ≤ 1 target; future studies linking quantitative TOF metrics and EMG readouts may clarify residual confounding between NMB depth and index behavior.

Fifth, we did not evaluate postoperative outcomes (e.g., delayed emergence, postoperative delirium, postoperative cognitive dysfunction); prospective studies should connect intraoperative index profiles to postoperative endpoints and test whether index-guided strategies (BIS-guided, PSI-guided, or trend-based multimodal protocols) improve recovery, hemodynamic stability, or neurocognitive outcomes.

Finally, PSI targets under remimazolam are not yet defined; cross-device mapping (e.g., BIS 40–60 vs. PSI 25–50) anchored to clinical endpoints and randomized comparisons are warranted to determine whether device-specific targets translate into better dosing accuracy and outcomes [25, 30–32].

## Conclusion

Under remimazolam anesthesia, BIS and PSI exhibited parallel temporal behavior, suggesting that both respond consistently to anesthetic depth despite using distinct algorithms. This finding provides practical reassurance that when either index departs from its target range, whether elevated or suppressed, the other generally trends in the same direction, reflecting shared cortical dynamics rather than isolated device artifacts. However, phase-specific discrepancies and broad limits of agreement indicate that these indices are not numerically interchangeable. Understanding this parallel yet non-identical behavior enables clinicians to interpret deviations of either monitor in context and apply each device appropriately within its algorithmic framework.

## Supplementary Information

Supplementary Material 1.

## Data Availability

The datasets generated and analyzed in the current study are not publicly available because they contain patient-level perioperative information collected at a single institution. De-identified summary data are available from the corresponding author upon reasonable request and with permission from the Institutional Ethics Committee of Hitachi General Hospital.

## Abbreviations

BIS: Bispectral Index
PSI: Patient State Index
EMG: electromyography activity (%)
SQI: signal quality index (%)
