# Supplementary material for: Comparison of bispectral index and patient state index during general anesthesia with remimazolam
Source: BMC Anesthesiol. 2025 Nov 17;25:566. doi: 10.1186/s12871-025-03464-6 (PMC12625464; doi:10.1186/s12871-025-03464-6)
Supplement: Supplementary file 1 — Supplementary Material 1. [file 12871_2025_3464_MOESM1_ESM.docx]

**Supplementary materials**

**Supplementary Table S1. Timecourse summary of BIS and PSI at predefined time points**

| **Time point** | **n** | **BIS (mean ± SD)** | **PSI (mean ± SD)** |
| --- | --- | --- | --- |
| T0 | 60 | 93.5 ± 9.4 | 90.8 ± 6.6 |
| T1 | 60 | 73.6 ± 15.1 | 68.3 ± 16.5 |
| T2 | 60 | 49.3 ± 10.3 | 37.8 ± 8.7 |
| T3 | 60 | 51.3 ± 10.3 | 41.1 ± 9.5 |
| T4 | 60 | 50.1 ± 10.3 | 40.2 ± 8.8 |
| T5 | 60 | 48.5 ± 9.5 | 38.8 ± 9.5 |
| TI | 60 | 48.0 ± 9.0 | 37.5 ± 8.0 |
| T15 | 60 | 46.2 ± 9.1 | 35.5 ± 9.1 |
| T30 | 60 | 46.4 ± 9.1 | 34.6 ± 8.0 |
| T45 | 60 | 47.6 ± 9.3 | 34.9 ± 8.0 |
| T60 | 54 | 48.0 ± 9.0 | 34.6 ± 7.8 |
| T75 | 46 | 47.7 ± 10.5 | 33.6 ± 8.7 |
| T90 | 38 | 48.5 ± 9.2 | 35.5 ± 7.3 |
| T105 | 30 | 50.0 ± 11.8 | 35.8 ± 9.6 |
| T120 | 28 | 48.9 ± 9.9 | 35.6 ± 6.6 |
| T135 | 24 | 49.2 ± 9.4 | 36.3 ± 7.7 |
| T150 | 22 | 50.7 ± 10.3 | 38.1 ± 7.8 |
| T165 | 17 | 50.2 ± 8.5 | 37.3 ± 7.1 |
| T180 | 15 | 48.4 ± 9.3 | 36.2 ± 6.4 |
| TE | 60 | 88.3 ± 12.3 | 75.9 ± 11.6 |
| TX | 60 | 93.5 ± 6.2 | 80.5 ± 12.1 |

*Abbreviations: BIS, Bispectral Index; PSI, Patient State Index; SD, standard deviation; LOC, loss of consciousness; TE, tracheal extubation; TX, leaving operating room.*

*Time points: T0=Awake, T1=LOC, T2=Intubation, T3–T5=Pre-incision, TI=Skin incision, T15–T180=every 15 min after incision, TE=Tracheal extubation, TX=Leaving operating room. The sample size (n) may decrease at later time points, depending on the case duration.*

**Supplementary Table S2. Tukey-adjusted pairwise comparisons of BIS–PSI differences across anesthetic phases within EMG strata**

| EMG | Contrast (phase) | Estimate  (ΔEMM) | P (Tukey) |
| --- | --- | --- | --- |
| Low | Awake - LOC | -4.4 | 0.996 |
| Low | Awake - Intubation | -10.7 | 0.829 |
| Low | Awake - Preincision | -8.6 | 0.923 |
| Low | Awake - Intraoperative | -11.6 | 0.768 |
| Low | Awake - Emergence | -9.0 | 0.927 |
| Low | LOC - Intubation | -6.3 | 0.054 |
| Low | LOC - Preincision | -4.1 | 0.270 |
| Low | LOC - Intraoperative | -7.1 | 0.002 |
| Low | LOC - Emergence | -4.6 | 0.865 |
| Low | Intubation - Preincision | 2.1 | 0.684 |
| Low | Intubation - Intraoperative | -0.9 | 0.987 |
| Low | Intubation - Emergence | 1.7 | 0.998 |
| Low | Preincision - Intraoperative | -3.0 | 0.002 |
| Low | Preincision - Emergence | -0.5 | 1.000 |
| Low | Intraoperative - Emergence | 2.5 | 0.982 |
| High | Awake - LOC | 2.2 | 0.908 |
| High | Awake - Intubation | -5.2 | 0.961 |
| High | Awake - Preincision | -5.1 | 0.588 |
| High | Awake - Intraoperative | -3.2 | 0.757 |
| High | Awake - Emergence | -6.9 | <0.001 |
| High | LOC - Intubation | -7.3 | 0.851 |
| High | LOC - Preincision | -7.2 | 0.851 |
| High | LOC - Intraoperative | -5.3 | 0.292 |
| High | LOC - Emergence | -9.1 | <0.001 |
| High | Intubation - Preincision | 0.1 | 1.000 |
| High | Intubation - Intraoperative | 2.0 | 1.000 |
| High | Intubation - Emergence | -1.7 | 1.000 |
| High | Preincision - Intraoperative | 1.9 | 0.993 |
| High | Preincision - Emergence | -1.8 | 0.990 |
| High | Intraoperative - Emergence | -3.7 | 0.483 |

*Legend: Data are presented as estimated marginal mean (EMM) contrasts from a linear mixed-effects model (LME) evaluating the interaction between anesthetic phase and electromyography (EMG) activity.*

*Pairwise comparisons were adjusted using the Tukey method.*

*Positive values of ΔEMM indicate higher BIS relative to PSI.*

*Simultaneous confidence intervals were not computed; inference is based on Tukey-adjusted p values.*

*These results identify phase-to-phase changes in BIS–PSI differences within each EMG stratum, complementing the EMM summary in Table 5 and the graphical trends in Figure 4.*

*Abbreviations: BIS = Bispectral Index; PSI = Patient State Index; EMG = electromyography activity; EMM = estimated marginal mean; LME = linear mixed-effects model.*

**Supplementary Table S3. Tukey-adjusted pairwise comparisons of BIS–PSI differences across anesthetic phases (EMG-averaged main effect)**

| Contrast (phase) | Difference [95% CI] | P (Tukey) |
| --- | --- | --- |
| LOC-Awake | 2.6 [-2.1–7.4] | 0.615 |
| Intubation-Awake | 8.9 [4.2–13.7] | <0.001 |
| Preincision-Awake | 7.3 [3.4–11.2] | <0.001 |
| Intraoperative-Awake | 9.9 [6.3–13.4] | <0.001 |
| Emergence-Awake | 10.1 [6.0–14.2] | <0.001 |
| Intubation-LOC | 6.3 [1.5–11.1] | 0.002 |
| Preincision-LOC | 4.7 [0.8–8.6] | 0.008 |
| Intraoperative-LOC | 7.2 [3.7–10.8] | <0.001 |
| Emergence-LOC | 7.5 [3.3–11.6] | <0.001 |
| Preincision-Intubation | -1.6 [-5.5–2.3] | 0.834 |
| Intraoperative-Intubation | 0.9 [-2.7–4.5] | 0.979 |
| Emergence-Intubation | 1.2 [-3.0–5.3] | 0.968 |
| Intraoperative-Preincision | 2.5 [0.3–4.8] | 0.017 |
| Emergence-Preincision | 2.8 [-0.3–5.9] | 0.101 |
| Emergence-Intraoperative | 0.2 [-2.4–2.9] | 1.000 |

*Legend: Data are presented as pairwise comparisons of estimated marginal means (EMMs) for BIS–PSI differences across anesthetic phases, derived from a linear mixed-effects model (LME) that included anesthetic phase as a fixed effect.*

*Comparisons were adjusted using the Tukey method.*

*Positive values indicate higher BIS relative to PSI.*

*These results summarize the overall phase-dependent pattern of BIS–PSI differences averaged across EMG levels, complementing the stratified analyses shown in Table 5 and Supplementary Table S2.*

*Abbreviations: BIS = Bispectral Index; PSI = Patient State Index; EMM = estimated marginal mean; LME = linear mixed-effects model; CI = confidence interval.*
